# Varietal Screening of Durum Wheat Varieties for Resistance to *Pyrenophora tritici-repentis* (Tan Spot) under Field Conditions

**DOI:** 10.1155/2022/6433577

**Published:** 2022-05-27

**Authors:** Salma Tissaoui, Marwa Hassine, Amira Mougou-Hamdane, Ala Eddine Ben Araar, Romdhane Nasraoui, Bouzid Nasraoui

**Affiliations:** ^1^LR14AGR02, Laboratory of Bio-Aggressors and Integrated Pest Management in Agriculture, National Agronomic Institute of Tunisia, University of Carthage, Tunis, Tunisia; ^2^LR14AGR01, Laboratory of Genetics and Cereal Breeding, National Agronomic Institute of Tunisia, University of Carthage, Tunis, Tunisia; ^3^National Institute of Field Crops (INGC) Boussalem, Tunisia

## Abstract

Tan spot disease caused by *Pyrenophora tritici-repentis* was becoming more bred in Tunisia during the last decade. The search for resistant varieties against the increased virulence diversity of *P. tritici-repentis* is presently considered as a priority. Seven of the most commercialized durum wheat varieties in Tunisia (cvs. Maâli, Salim, Razzak, Monastir, Khiar, Inrat100, and Sculptur) were inoculated with five characterized fungal strains under field conditions, during two seasons. The variance analysis revealed that strains Ech8F_6_ and B4.8 used in inoculation are the most virulent ones. These strains hosting *ToxB* gene caused chlorosis symptom on the tested varieties. The other strains induced necrosis with yellow halo and host *ToxA* gene were less virulent. The area under disease progress curve values revealed that Maâli is the most vulnerable genotype compared to the new selected varieties Monastir and Inrat100. A variable tolerance rate of the varieties to tan spot disease was also highly visible on yield components. The losses were about 22.2% of the thousand kernel weight in Maâli variety, 35% of spikes/m^2^ in Inrat100 variety, 32.5% of kernel number/spike, and 25.2% of yield grain in Monastir variety. This effect evaluation of the strains harbouring *ToxA* and *ToxB* genes could be responsible for the identification of potentially susceptible genes *Tsn1* and *Tsc2* representing resistance sources for breeding programs.

## 1. Introduction

Tan spot disease, caused by the Ascomycete *Pyrenophora tritici-repentis* (Died.) Drechs. (anamorph: *Drechslera tritici-repentis*) (Died.) Shoem.), is a foliar disease of durum wheat ([1]; Tadesse et al., [2]) and bread wheat [3] in many wheat producing areas worldwide [4]. This necrotrophic fungal pathogen *P. tritici-repentis* (Ptr) occurs in warm and temperate wheat growing regions [5] as Tunisia [6]. It induces up to 53% of yield losses due to the reduction of photosynthetic area of leaves [7–9]. The disease is also called yellow spot [7] because of the oval or diamond necrotic lesions surrounded by chlorotic borders or yellowish haloes developed on susceptible wheat plants [7, 10], giving it a distinctive “eye-spot” appearance [11]. The climate change which is the origin of the development of severe disease epidemic could lead to an important reduction of kernel weight, number of grains by head, total biomass [1, 12], and grain quality by the induction of red and dark smudge symptom [13, 14]. The increase of yield losses was also associated to the overwinters in wheat stubble in the field, cultural practices, monoculture, susceptible cultivars, and conservation agriculture [3, 15]. These different practices with the reproductive cycle of the fungi during the season resulted into a large genetic diversity [16]. Therefore, this causal agent was characterized by a virulence variability [17] and distinct symptoms type (chlorotic or necrotic) on four genotypes Glenlea, 6B365, 6B662, and Salamouni (wheat differential set) [18–21]. This virulence variability was associated to eight races of Ptr [17]. The interaction of Ptr-wheat pathosystem follows in general an inverse of the gene-for-gene model [14] and race-specific [22]. The distinct races have the ability to produce three host selective toxins (HSTs) known as PtrToxA, PtrToxB, and PtrToxC, which are considered pathogenic factors. Toxin can be produced singularly, e.g., Ptr ToxA, Ptr ToxC, or Ptr ToxB, by strains of races 2, 3, and 5, respectively, or in combination of two toxins as Ptr ToxA+Ptr ToxC, Ptr ToxB+Ptr ToxC, and Ptr ToxA+Ptr ToxB, by each of the race strains 1, 6, and 7, respectively. In addition, all three toxins may be produced simultaneously by strains of race 8. However, no known toxins are produced by strains of race 4; therefore, it does not have any pathogenic aptitude [3, 21]. The specific toxin Ptr ToxA induces necrosis symptoms in sensitive wheat genotypes [21] and influences severity of tan spot disease which depends on the host genetic ability [3, 23, 24]. The sensitivity reaction of wheat to Ptr ToxA toxin is resoluted by the Tsn1 gene [25], which is located on the long arm of chromosome 5BL [26]. *Tsn*1 gene interacts with Tox A effector gene in the pathogen, responsible of specific toxin Ptr ToxA synthesis (Zhang et al., [27]). Their interaction has a little contribution to the installation of the disease on durum wheat [28, 29] but plays an important role in the disease development in bread wheat [30, 31]. The protein toxin Ptr ToxB [21] is controlled by the single dominant gene *Tsc*2 located in the short arm of chromosome 2B in host [32]. The Tsc2-Ptr ToxB interaction was found to have an important role in tan spot progress on bread wheat [30, 31] and durum wheat [28, 29]. It could eventually contribute to about 69% of the symptom's variation caused by race 5 ([33], Faris et al., [34]). The nonionic and polar molecule Ptr ToxC is controlled by the insensitivity gene *Tsc*1 which is located in the short arm of chromosome 1A [35]. In Tunisia, the distinct profile of pathogen effector genes [6, 36], and several races were discovered [6, 37] in strains collected from different areas of Northern Tunisia. Prevalence, incidence, and severity percentages varied between geographical region and cultivated varieties [38, 39] but the response of commercialized wheat varieties to tan spot pathogen, and their resistance level remains unknown. The objectives of the study are (i) to evaluate the severity and the progress of the disease caused by inoculation under field conditions and (ii) to assess the resistance levels of the most used Tunisian durum wheat varieties to distinct strains of pathogen.

## 2. Materials and Methods

### 2.1. Experimental Test

#### 2.1.1. Test Site

**Figure 1:**
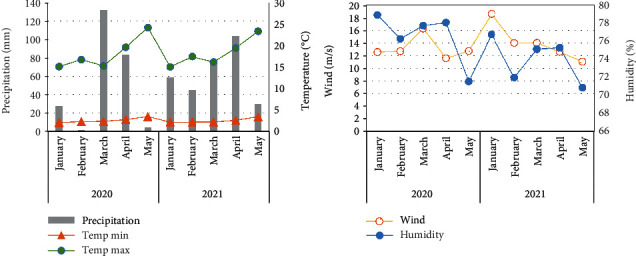
Monthly averages of the various meteorological parameters: Temperature, precipitation, humidity, and wind speed during 2020 and 2021 seasons.

The trial was conducted during 2019-2020 and 2020-2021 wheat-growing seasons, in the northern parts of Tunisia at the experimental station of the National Institute of Field Crops (INGC) at Oum Heni region (37°05′00 N 9° 50′49 E), Governorate of Bizerte. The experimental site is located at 112 m above sea level, 5.8 km from the Bizerte Lake, and 10.5 km from Ichkil Lake. This area is part of a subhumid bioclimatic zone and characterized by an average annual pluviometry that ranges between 600 and 800 mm. Temperature, precipitation, humidity, and wind speed were recorded during the study period (Figure 1).

#### 2.1.2. Trial Management and Plant Materials

The field experiment was performed in a split-splot system in a randomized complete block with three replications. Each of the subplot experiment measured 2 m × 1.5 m with 0.50 m spacing, and blocks were separated by an alley 1.50 m wide. Sowing was carried out at the rate of 120 kg/ha. During 2019-20, the trial was established at November 18^th^ 2019, while during 2020-2021, it was at December 17^th^ 2020. Seven Tunisian durum wheat varieties (cvs. Salim, Maâli, Razzak, Monastir, Khiar, Inrat 100, and Sculptur) were used in the present study based on their susceptibility levels to tan spot, *Septoria tritici* blotch and yellow rust. Fertilizers (N, P_2_O_5_, and K_2_O) and herbicides were applied to ensure adequate crop development at tilling and stem stage (Z31 and Z56).

### 2.2. Isolation, Identification, and Effector Gene Characterization of *P. tritici-repentis* Strains

#### 2.2.1. Pathogen Isolation

Isolation was performed on wheat leaves showing tan spot symptoms, collected from different Tunisian infested fields. The infected leaves were cut into small pieces, sterilized in sodium hypochlorite solution (3%) during 3 min, and were rinsed thrice in sterile distilled water during 5 min, then placed in Petri dishes containing humidified filter paper. The dishes were incubated in a moist chamber (intense light for 18 h at 20°C followed by 6 h at 15°C in the dark) for 72 h to induce conidia production. After incubation, leaf pieces were examined using a binocular stereomicroscope and a single conidium of the fungus was extracted using a steel needle and transferred to PDA (Potato Dextrose Agar) or V8-PDA medium (agar 20 g, glucose 20 g, CaCO_3_ 3 g, V8-juice 150 ml, and 850 ml boiling potato). Microscopic observation was performed in order to check the sole conidium development. After verification, single-spore cultures were incubated in the darkness at 20°C for 7 days [40, 41]. Culture plates were used subsequently for DNA extraction and PCR identification.

#### 2.2.2. DNA Pathogen Extraction

DNA extraction concerned 63 fungal mycelium of 7 days-old grown on PDA media plates derived from single spore. Each fungal mycelium was carefully scrapped off and was harvested in 1.5 ml Eppendorf tube, using mix protocols of Leeand Taylor [42] and that of Mironenko et al. [43]. Then, volume of 600 *μ*l of CTAB 2% (cetyltrimethylammonium bromide) extraction buffer was added to Eppendorf tubes which were heated at 60°C for two hours and well vortexed every 15 min. One volume of chloroform/isoamyl alcohol (24 : 1*v*/*v*) was added and vortexed and then centrifuged at 10.000 g for 10 min. The aqueous phase, containing the DNA, was transferred to new Eppendorf tubes by adding isopropanol volume and let precipitate overnight [44]. The pelleted DNA was washed twice with ethanol 70% and dried at room temperature. Dried DNA was dissolved in 80 *μ*l of sterile distilled water and then analyzed using an QIAxpert system (QIAGEN, QIAxpert) to control the gDNA quality and to quantify its concentration.

#### 2.2.3. Pathogen Identification by Specific Primers

A primer pair DTR1-F (5′- ACCAATATGAAGCCGGACTG-3′) and DTR1-R (5′-CTCGGGAGAGAGACAAGACG-3′) were used for specific PCR identification of *P. tritici-repentis* [45]. PCR were performed in 25 *μ*l total volume containing RNAase free water, 20 ng/*μ*l genomic DNA, 10x complete buffer, 10 Mm DNTP mix, 10 *μ*M each forward and reverse primer, and 0.5 *μ*l of Taq polymerase (5 units/*μ*l) [46]. Amplification was performed using the following PCR program: initial denaturation at 94°C for 1 min, 35 cycles at 94°C for 30 s, 60°C for 30 s, 72°C for 1 min, and extra extension at 72°C for 5 min. Electrophoretic detection of PCR products was performed in 1.4% agarose gel stained with ethidium bromide and photographed under UV light [46].

#### 2.2.4. Molecular Characterization for *ToxA* and *ToxB* Genes

The DNA was extracted from 63 strains for the detection of the effector genes *ToxA*, *ToxB*, and *toxb* genes. The gDNA were amplified by PCR to detect effector genes *ToxA*, *ToxB*, and their homologue *toxb* as per the protocol described by Andrie et al. [47] with a slight modification. Then, a multiplex PCR was performed on the same DNA to confirm the result of PCR analysis, using specific primer pairs and amplification conditions were as described by Tissaoui et al. [48]. All the PCR products were composed in LTCG-A22 LABTRON Therma cycler and thereafter analyzed by gel electrophoresis through 1.5% agarose gel in 1x TAE buffer after staining with ethidium bromide dye. The sizes of the PCR amplicons were estimated against 1 kb plus ladder (Grisp, GRS Ladder) and visualized under UV light.

### 2.3. Inoculation Trial

#### 2.3.1. Inoculum Preparation

Ptr strains were grown on V8-PDA agar (150 ml V8 juice, 20 g agar, 10 g PDA, 3 g CaCO_3_, and 850 ml distilled water) in the dark at 20°C for 6 days. The plates were filled with sterile distilled water to scrap the mycelium; then, excess water was poured off. The plates were incubated under continuous light at 20°C for 48 h followed by 24 h in dark at 15°C to induce conidia production (Balance et al., [41, 49]). After the final incubation period, plates were examined with binocular stereomicroscope to check the conidial production. Conidia was harvested by flooding the plate with 10 ml of sterilize distilled water and gently brushing to pluck off the conidia from conidiophores. The resulting conidial suspension was adjusted to 4 × 10^3^ conidia/ml using a hemocytometer (Neubauer hemocytometer) and an optical microscope. Two drops of Tween 20 (per 250 ml) were added to conidial suspension as a surfactant, based on the procedure described by Ali and Francl [50], Lamari and Bernier [51], and Moreno et al. [44].

#### 2.3.2. Inoculation

The virulence of 63 strains was tested firstly at seedling stage in a growth chamber on fourteen durum wheat genotypes. The classification of the strains was based on their cultural and microscopic morphology, pathogenic analysis, and effectors genes as described by Tissaoui et al. [39], Tissaoui et al., [37]. Five strains of Ptr showed a high genotype-strain interaction and virulence on most of the tested durum wheat germplasm and were therefore chosen for field inoculations. All used wheat varieties have been inoculated at GS 32 and GS 33 [52], with the five selected strains: Ech8F_6_, 103 F_1_, J4.2ù, 67.11, and B4.8 (Table 1) using a modified protocol described by Evans et al. [53] twice with 15 days interval. Inoculum was prepared, and concentration was adjusted to 4000 conidia ml^−1^[54] for spraying by a backpack pressure sprayer (16.8 l). The control plots were inoculated with water. The inoculation of plots has been proceeded by a rain for 48 h and during climatic conditions characterized by temperature ranged from 12 to 15°C, which is required for tan spot infection.

### 2.4. Disease Assessment

Disease symptoms were evaluated visually based on the necrotic leaf area and /or chlorosis areas from the uppermost fully developed four leaves of thirteen marked plants for each split-plot [53]. Disease severity was scored using the double-digit scale (00-99) developed as a modification of Saari and Prescott's severity scale, to assess wheat foliar diseases (Eyal and Prescott, [55, 56]). The first score (*D*1) indicates vertical disease progress on the infected plants, which varies from 0 to 9 with ‘5' indicating that the mid-point of the plant and ‘9' denoting the presence of the disease on the spikes [18]. The second digit (*D*2) refers to the severity at the leaf scale. A global percentage was determined using the formula of Sharma [57]: Severity percentage (%) = (*D*1/9)(*D*2/9)∗100.

A continuous consecutive evaluation was realized on marked plants at a weekly basis, from inoculation until the onset of tan spot symptoms at leaf scale. The disease progress of tan spot was carried out during 2019-2020, at three dates, while six evaluations were carried out during 2020-21. Therefore, as soon as the symptoms appeared, the ratings of disease severity started visually on different crop stages as stem elongation, flag leaf booting, heading, and flowering on the marked plant. These different stages were considered critical for grain yield production, especially the reduction of the green leaf area on the flag leaf during this period which may result in the most significant yield losses [58]. The obtained disease rating during the two growing seasons was evaluated by area under disease progress curve (AUDPC) for each wheat cultivar calculated using the estimated severity percentage in accordance with days interval following formula [59]:

(1) $$\begin{array}{c} \text{AUDPC}=\overset{n-1}{\underset{i=1}{\sum }}\frac{y_{i}+y_{\left(i+1\right)}}{2}X\left(t_{\left(i+1\right)}-t_{i}\right), \end{array}$$

where *y*_*i*_ is the tan spot severity at time *t*_*i*_, *t*_(*i* + 1)_ − *t*_*i*_ is the time interval (days) between two disease scores, and *n* is the number of times when tan spot was recorded.

### 2.5. Yield Parameter Assessment

At maturity, 30 plants were randomly selected per replicate in individual manually collected plot, and total spikes number per m^2^ was evaluated (S/m^2^). The spikes were threshed in order to determine grain number per spike (GN/S), thousand kernel weight (TKW), and grain yield (GY) which represents the grain weight on a plant-by-plant basis ([60]; Pandey et al., [61]).

### 2.6. Statistical Analysis

Variance analysis (ANOVA) was conducted on severity and yield components data using SAS software version 9.4. Means were estimated and significance of difference (*P* < 0.05) between means was determined by Fisher's Test. To extract information from effect of inoculation on wheat genotypes, principal component analysis (PCA) was performed based on Pearson correlation coefficient. The analysis used mean scores for each variety, to identify relationship between AUDPC, Spike/m^2^, GN/S, TKW, and GY, and was conducted using SPSS software (IBM, SPSS, Statistics, version 23.0.0.0).

## 3. Results

### 3.1. Characterization of Strains

**Figure 2:**
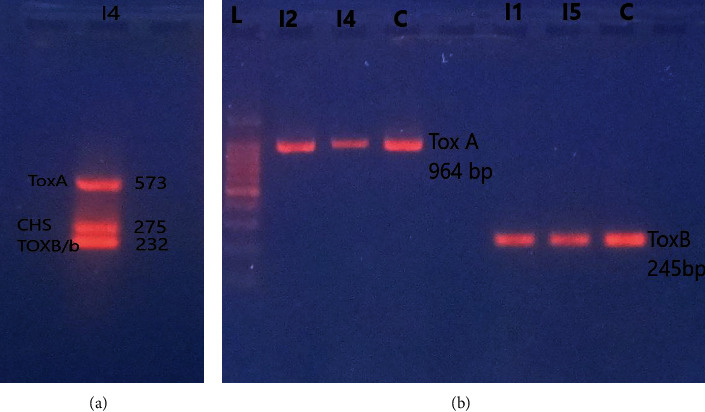
PCR amplification with primers specific for *ToxA*, *ToxB*, and *Toxb* genes of *Pyrenophora tritici-repentis*. (a) A multiplex PCR with specific primers to *ToxA*, *ToxB*, *Toxb*, and CHS1 gene. (b) A singleplex PCR with specific primers to *ToxA* and *ToxB* genes. L: ladder 100 bp; I1; I2, I4, I5: tested strains; C: control; CHS1: chitin synthase 1 gene used as internal control for the presence of fungal DNA.

DNA amplification of tested strains was realized with simplex PCR to detect effector genes *ToxA*, *ToxB*, and the homolog *toxb*. PCR result revealed that CHS-1 gene was amplified from all strains. I1 and I3 strains possess *ToxB* gene corresponding to the size amplicon of 245 bp and its homolog *toxb*, but only *ToxB* gene in I5. The strains I2 has harbored both effector genes *ToxA* (corresponding to size band of 964 bp) and *ToxB* genes (245 bp) (Figure 2(b)). The multiplex PCR amplification which was used to verify the result of simplex PCR has detected *ToxA* (573 bp), *ToxB* (232 bp), and t*oxb* (232 bp) genes in I4 (Figure 2(a)). These results indicated distinct pathotypes of Ptr strains based on their hosting distinct effector genes.

### 3.2. Severity Assessment

The data analysis was performed using ANOVA while monitoring the effect of the year and the wheat variety during the seasons 2019-2020 and 2020-2021. It is aimed at evaluating the resistance of wheat varieties to Ptr under field conditions using artificial inoculation. The most observed lesion type was necrosis surrounded by yellow halo designed by necrosis with chlorosis. Chlorosis symptom was the least recorded during the experiment on the different tested durum wheat varieties. These typical symptoms were developed due to the distinct pathotypes corresponding to the amplification of different effector genes. The minor profile was detected for both *ToxB* and *ToxA* genes responsible of necrosis with chlorosis symptom on varieties cvs. Salim, Khiar and Maâli, during 2019-2020 and 2020-2021 seasons (Table 2).

**Figure 3:**
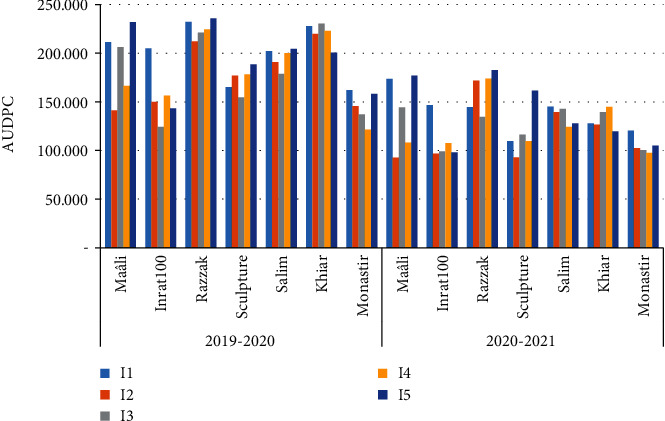
AUDPC rate of seven durum wheat varieties in reaction to inoculation with all *Pyrenophora. tritici-repentis* strains under field conditions during 2019-2020 and 2020-2021 seasons.

The strains I2 and I4 which contain *ToxA* gene induced necrosis with chlorosis (typical symptom) in the presence of *ToxB* gene, encoding the toxins Ptr ToxA and Ptr ToxB, respectively. These strains have most likely caused a gene susceptible reaction to the combination of necrotrophic effector *ToxA* and *Tox B* genes in the concerned varieties. Wheat varieties cvs. Khiar and Salim could possess a dominant *Tsn1* or recessive *tns1* susceptibility gene to the specific toxin Ptr ToxA. Other strain I5 induced chlorosis symptoms on wheat varieties e.g. cvs. Maâli, Razzak, and Salim and produced the HST Ptr ToxB, probably stimulated reaction of Tsc2 gene of sensitivity in the host. Susceptibility to the strain I5 (*Tox B* gene possessor) was higher in cv. Inrat100 (16.5%) and cv, Razzak (4.7%) than cv. Monastir (Table 3). The susceptibility reaction to I4 was higher in cv. Inrat100 (24.8%) and cv. Khiar (21.5%) than cv. Monastir, which was probably due to two effector genes in pathogen and two sensitivity genes in the wheat variety interactions. According to the recorded severity on all wheat varieties, the distinct strains were clustered in two classes (Figure 3).

The AUDPC values were significantly affected by the year, the variety susceptibility, and the distinct strain of the pathogen in the experiment. A significant difference in the reaction of the tested wheat varieties to the inoculation with Ptr was observed during the two seasons (2019-2020 and 2020-2021) (Figure 3). The AUDPC values during 2020-2021 decreased with an average from 40.76 to 66.37% compared to the previous season 2019-2020. The highest AUDPC values were recorded during 2019-2020 for all varieties and strains. The lowest AUDPC values were recorded with the varieties cvs. Inrat100 and Monastir during 2019-2020 and in cv. Sculptur during 2020-2021. The highest AUDPC values were observed with the varieties cvs. Razzak and Maâli during 2019-2020 (about 200) and cv. Razzak (134.55) during 2020-21 season with a decrease of 50.9%. During the two seasons, cvs. Salim and Khiar showed a medium AUDPC values (180.3, 113.22) and (154.1, 109.73), respectively, during 2019-2021 seasons. During the two seasons, strains I1 and I5 induced the highest AUDPC values, which was 16% higher in severity when compared to the others strains with the all tested wheat varieties. Therefore, these strains (I1 and I5) are the most pathogenic, harboring ToxB gene which had been probably reacting with the sensitivity gene *Tsc2* in durum wheat varieties. However, strain I3 was the less virulent strain on the tested varieties during the two seasons, despite its production of Ptr ToxB toxin; it could be due to the genetic sensitivity of cv. Inrat100, e.g., the least virulent strain is I2 which induced little typical symptom on cv. Sculptur, and the least AUDPC values which decreased with 35.71% and 52.63% in cvs. Maâli and Sculptur, respectively, from 2019-2020 to 2020-2021.

### 3.3. Strain Effect on Yield Components

The ANOVA analysis indicated at *p* ≤ 0.01 significant genotypic differences in the effects on the yield components such as thousand kernel weight (TKW), spike number per m^2^, kernel number per spike, and grain yield in the artificial inoculation under field conditions (Table 4).

#### 3.3.1. TKW

ANOVA analysis indicated at *p* < 0.05 a significant genotypic difference in the effect on thousand kernel weight (TKW) for all tested varieties during the two seasons. TKW variability was due to the different resistance levels among wheat varieties to the tan spot pathogen. During the two season experiment, the majority of the varieties did not show a significant difference of TKW values between treatment and control test, except for two varieties, cvs. Maâli and Inrat100 (Table 4). In fact, cv. Maâli recorded about 22.22% losses of this parameter caused by I1 strain, during the 2020-2021 season and cv. Inrat100 had lost about 24% of TKW due to I1, I4, and I5, during the 2019-2020 season. These strains as I1 and I5 are responsible of the production of toxin Ptr ToxB specific in the activation of the sensitivity gene Tsc2, while I5 could stimulate Tsn1-ToxA and Tsc2-ToxB interactions in the different tested varieties because of two distinct virulence effector genes.

#### 3.3.2. Number of Spikes per m^2^

This parameter was significantly different at *p* ≤ 0.05 regarding the tested varieties, the strain^∗^wheat interaction and the compared seasons of experiment (Table 4). There was a loss in spikes/m^2^ varied from 16.64% to 35.13% for all varieties due to the inoculation of different pathogen strains. The lowest loss was recorded with cv. Razzak (19.64%) followed by cv. Maâli (21.4%) and was caused mainly by the strain I1. However, the highest losses of spikes/m^2^ (35.13% and 35%) were observed with cvs. Inrat100 and Sculptur due to the inoculation with strains I4 and I2, respectively. The average loss was obtained with cvs. Salim (24.63%) and Monastir (28.57%) caused by the effect of the inoculation with I5 and I1 strains, respectively.

#### 3.3.3. Number of Kernels per Spike

Significant differences at *p* ≤ 0.05 among the tested genotypes and in strain∗wheat interaction was recorded for the kernels per spike (Table 4). The highest number of kernels/spike (51.46) was noted during 2019-2020 with cv. Inrat100. Compared to the control, this parameter showed no significant difference with the inoculation treatment, except the inoculation with strain I4 which induced 20% loss of grains/spike. The lowest loss of kernel number per spike was observed with I2 inoculation for all varieties, where there was no significant difference compared to the control, except with cvs. Khiar and Monastir. The highest loss was recorded with cvs. Monastir (32.5%) and Khiar (26.1%) in reaction to the inoculation with I4 strain. This strain characterized by combinations of effector genes may be responsible of the activation of more than one sensitivity gene.

#### 3.3.4. Yield (q/ha)

After wheat harvesting, grain yield (q/ha) were estimated for each varieties and each individual plot (Table 4). There were significant differences between the seasons and between varieties in the same season. The yield loss was the highest during the first growing season compared to the second season. The lowest yield loss was recorded with cvs. Maâli (6%) and Razzak (5%) while the highest yield loss was observed with cvs. Monastir (28.2%). The strain I1 induced the lowest loss of the yield and I4 and I5 caused the highest losses. I1 strain caused the highest loss in cv. Monastir, and I2 induced the lowest loss in cv. Maâli. However, the least effect on yield was obtained for the varieties known for their high productivity as cvs. Salim and Sculptur.

### 3.4. Assessment of Relationship between AUDPC and Yield Components

**Figure 4:**
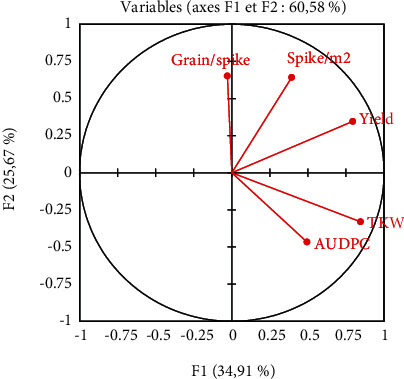
Biplot from principal component analysis.

**Figure 5:**
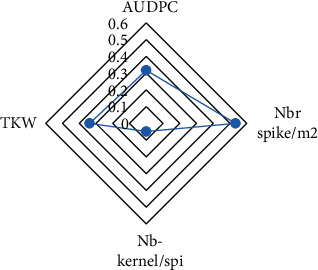
Chart of principal component analysis of the yield components and AUDPC.

Principal component analysis (PCA) of AUDPC and yield components, allowed detecting similarities in wheat varieties regarding tan spot responses during the growing seasons (Figure 4). The first two dimensions explained the correlated variability of the tested varieties, with inertia equal to 60.58%. Dimension 1 accounted for 34.91% and dimension 2 accounted for 25.67% of the data variance. The first component was represented by TKW, and the second component was correlated with the number of grains/spike. The analysis of AUDPC and yield components did not outcome an evident grouping. According to Figure 5, the kernel number/spikes had the lowest value (0.09) while the number of spikes/m^2^ had the highest value (0.5).

## 4. Discussion

The characterization by PCR multiplex of used strains has shown the presence of effector genes and their homologs. All the strains possess *ToxB* gene, only two strains possess *ToxA* gene, and three strains contain the homolog *Toxb*. These profiles of fungi were identified on investigation of Kamel et al. [6] and Laribi et al. [36] in Tunisia. These five tested strains of Ptr exhibited different levels of virulence on the most cultivated durum wheat varieties used by Tunisian farmers. This virulence variability of strains was mainly determined by host-specific toxins produced by Ptr strains which influenced the genetic response of durum varieties. The strains Ptr ToxB producer as I1 and I5 were the most virulent by probably reacting with sensitivity gene *Tsc2* in all tested varieties. This host reaction was reported by Ciuffetti et al. [62], with strains responsible of the production of Ptr ToxB controlled by the dominant sensitivity gene *Tsc2*. This same*ToxB-Tsc2* interaction was present in the Ptr-durum wheat populations used by Virdi et al. [63]. The gene *Tsc2* conditioning susceptibility to the Ptr ToxB toxin was also present in the durum wheat varieties as mentioned by Faris et al. [30]. However, the increasing effect of the virulence of this toxin (Ptr ToxB) as produced in the present study by the strains I1 and I5 on the commercial wheat varieties was mainly due to their prevalence in Tunisian climate condition as reported by Kamel et al. [6]. Same investigations in North Africa conducted by Benslimane et al. [64] and Gamba et al. [65] have also demonstrated the increasing of Ptr *ToxB*-producing strains was due to the prevalence of durum wheat cultivation. Therefore, the host resistance mechanisms of cvs. Salim, Razzak and Maâli was probably interacting directly or indirectly using the dominant genes as *Tsn1* and *Tsc2* to our two main host-selective toxins (HSTs) (Ptr ToxA and Ptr ToxB). This susceptibility reaction of durum varieties would be due to the recognition of dominant necrotrophic effectors of I1 and I5 with a sensitivity gene could contribute to compatibility reactions as described by Faris et al. [30] and observed in cvs. Maâli and Razzak by Kamel and Cherif [38]. This same compatibility reaction was detected in the juvenile stage to different strains of Ptr by Tissaoui et al. [48] and under filed conditions during successive years of investigation in Tunisia ([38]; Tissaoui et al., [39], Tissaoui et al. [37]). Otherwise, the recently introduced cvs. Monastir and Sculptur were less susceptible than the three previous cited varieties in our present research. These findings regarding the reaction of cv. Monastir was reported by Elfahem [66], Kamel and Cherif [38], and Tissaoui et al., [39], Tissaoui et al. [37]. The varied reaction of wheat varieties to strains could allow to susceptible varieties to differentiate virulence in strains better than the resistant varieties. Similarly, the variability in varieties was better detected with virulent strains than less virulent strains [67].

The different responses to inoculation and disease development during the two growing seasons was eventually affected by weather conditions on the most susceptible wheat varieties, which is in agreement with Jenns and Leonard [68]. Disease severity was more important during growth season 2019-2020, compared to the following season because of the difference in humidity, air temperature and rainfall. These research results was demonstrated by Kremneva et al. [22], which highlighted the effect of the higher average air temperatures and humidity on the susceptibility response of the most winter wheat varieties compared to drier years. These same weather factors have influenced the onset and development of the pathogen and the host susceptibility level to the disease in different studies of Fernandez et al. [69].

Moreover, the assessment of the development of this foliar disease using area under disease progress curve (AUDPC) has revealed that the response of the tested varieties have been basically depending on the distinct profiles of tan spot inoculum and the weather conditions. This same effect was observed by Evans et al. [53], Kader et al. [67], and Wegulo et al. [70] by using AUDPC in the evaluation of the response of different wheat varieties to inoculation under field conditions. These AUDPC values permitted also the identification of the susceptibility of Tunisian commercial varieties to pathogen strains, which is in accord with Kader et al. [71] results. However, the different levels of susceptibility between varieties to the pathogen had influenced the yield components. This effect was highlighted by Evans et al. [53], who explained the effect of the genetic background of wheat genotypes on yield component. In addition, the TKW of the tested Tunisian durum wheat was reduced due to the inoculation, the susceptibility of varieties, and the climate conditions of each growing seasons. Same finding was reported in Lithuania, where the reduction of TKW was about 73% due to the inoculums, conductive weather conditions for the pathogen, and cultivar susceptibility [72]. In fact, the CPA analysis has shown a positive correlation between AUDPC and TKW for all the tested varieties. Based on the disease severity and the yield components data, the AHC has contributed to identify two clusters of strains (most pathogenic and less pathogenic) and two classes of wheat varieties. This analysis was used by Kader et al. [71] to detect lineage effect of some strains on the disease epidemiology based on some traits essential for the resistance screening.

Consequently, the intensive uses of these varieties and the unsuitable agricultural practices such as monoculture with inappropriate crop rotations and the minimum tillage containing infested straw residues could lead to the increase of virulence capacity of Ptr in Tunisia. This variability of the pathogen virulence due to the cultural practices was mentioned by Kohli et al. [73]. Therefore, the incidence and the severity of the prevalent Tunisian strains of tan spot has been increased due to the virulence and the variability of the pathogen (Tissaoui et al., [39], Tissaoui et al. [37]). In fact, the characterization of the Ptr strains is an interesting information necessary for breeders in order to develop tolerant wheat genotypes to the different tan spot pathotypes or races. Hence, the assessment of wheat genotype susceptibility using phenotypic screenings and molecular markers method is more efficient for genotype selection as described by Faris et al. [30]. The identification of the host resistance levels in this trial could contribute for the guidance of the breeding programs and the improvement of a sustainable management method to better control the tan spot disease.

## 5. Conclusion

The response of the most commercialized wheat varieties in Tunisia to the inoculation with five Ptr distinct strains varied between two season trials. Analysis of disease severity and yield components showed significant effects of varieties, strains, and their interaction. The high AUDPC allowed identifying the increasing of susceptibility of the commercial varieties to the pathogen strains. These high-interaction responses could explain the results and provide relevant information about their genetic background. This important interaction indicates that the varieties contain sensitive genes *Tsc2*. Moreover, phenotypic screening resistance evaluation allowed us detecting the two most virulent strains of Ptr harboring *ToxB* effector genes by inducing two symptoms of chlorosis and necrosis surrounded by a yellowish halo in the leaf host. This finding has of great importance for the identification of sources of tolerance to tan spot in order to enhance the development of varieties that are adapted to the increasing virulence of the pathogen strains under field conditions.

## Figures and Tables

**Table 1:** Origin and characterization of used strains of *Pyrenophora tritici-repentis* inoculated to durum wheat in field trial.

Strain	Code	Region	Year
I1	Ech8F_6_	Bizerte	2017
I2	103 F_1_	Manouba	2018
I3	J4.2	Jendouba	2019
I4	67.11	Nabeul	2017
I5	B4.8	Beja	2018

**Table 2:** Reactions of the most commercialized durum wheat varieties to the inoculation with the tested five strains of *Pyrenophora tritici-repentis*.

Strain	Tox gene	Reaction	Genotype	Symptom
I1	*ToxB+Toxb*	Necrosis+ chlorosis	Razzak	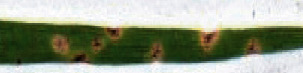
I2	*ToxA+ToxB*	Necrosis+yellow halo	Salim	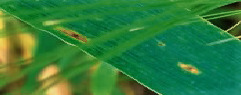
I3	*ToxB+Toxb*	Necrosis+yellow halo	Sculptur	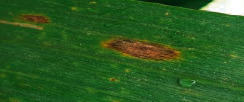
I4	*ToxA+ToxB+Toxb*	Necrosis+chlorosis	Khiar	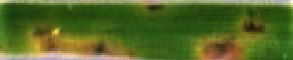
I5	*ToxB*	Chlorosis	Maâli	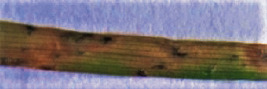

**Table 3:** Response of most commercialized durum wheat varieties to the artificial infection by *P. tritici-repentis* under the field conditions during the two growing seasons 2019-2020 and 2020-2021.

Strains	Maâli	Inrat100	Razzak	Sculptur	Salim	Khiar	Monastir
Severity assessment during 2019-2020							
I1	70.5 a B	70.8 a B	80.6 a A	77.3 a AB	80.6 a A	71.5 b B	70.6 ab B
I2	64.0 ab C	74.0 a AB	74.0 a AB	67.3 a BC	80.6 a A	73.8 b AB	64.0 b C
I3	60.6 b B	77.2 a A	73.8 a A	70.6 a A	70.6 b A	74.3 b A	74.1 a A
I4	63.8 ab B	80.6 a A	74.0 a AB	70.6 a AB	71.5 b AB	80.5 a A	67.3 ab B
I5	67.5 ab C	70.6 a BC	77.1 a AB	77.3 a AB	74.1b ABC	80.6 a A	70.6 ab BC
Severity assessment during 2020-2021							
I1	70.5 ab BC	70.5 b BC	80.5 a A	77.1 a BA	80.5 a A	70.5 a BC	64.0 a C
I2	63.8 b CD	70.5 b BC	80.5 a A	67.1 a BCD	73.8 a BA	73.8 a BA	60.5 a D
I3	74.1 a A	70.5 b A	70.5 b A	70.6 a A	73.8 a A	77.3 a A	60.5 a B
I4	67.1 ab BC	80.5 a A	70.5 b BC	67.1 a BC	73.8 a BA	80.6 a A	60.5 a C
I5	71.0 AB AB	80.5 a A	77.5 a A	77.3 a AB	73.8 a AB	70.6 a AB	67.1 a B

Means having the same letter within a column (lower case) and row (upper case) did not differ significantly by pairwise difference (*α* = 0.05).

**(a):** 

Sources of variation	TKW				*N* spikes/m^2^			
	Sum sq	Mean sq	*F* value	Pr (>*F*)	Sum sq	Mean sq	*F* value	Pr (>*F*)
Variety	4473.197	745.592	26.51	<.0001	226198.6	37699.77	6.55	<.001
Strain	302.9927	60.598	2.15	0.0615	124177	24835.4	4.32	0.001
Year	1168.102	1168.1	41.35	<.0001	1362.185	1362.18	0.24	0.627
Variety^∗^year	1061.811	176.968	6.29	<.0001	147979.2	24663.19	4.29	0.0005
Isolate^∗^year	222.9431	44.588	1.59	0.0166	31830.32	6366.06	1.11	0.358
Variety^∗^strain	1253.985	41.799	1.49	0.062	558902.8	18630.09	3.24	<.0001
Variety^∗^isolate^∗^year	1723.746	57.458	2.04	0.0024	403680.8	13456.02	2.34	0.0004

**(b):** 

Sources of variation	*N* kernel/spike				Yield			
	Sum sq	Mean sq	*F* value	Pr (>*F*)	Sum sq	Mean sq	*F* value	Pr (>*F*)
Variety	7594.958	1265.82	53.97	<.0001	2592.17	432.028	8.65	<.0001
Isolate	453.649	90.729	3.87	0.0024	1688.793	337.758	6.76	<.0001
Year	42.8868	42.8868	1.83	0.1781	4606.483	4606.48	92.18	<.0001
Variety^∗^year	125.054	20.8423	0.89	0.504	2020.987	336..831	6.74	<.0001
Isolate^∗^year	120.3299	24.0659	1.03	0.404	1767.586	353.517	7.07	<.0001
Variety^∗^isolate	1940.064	64.6687	2.76	<.0001	7281.568	242.718	4.86	<.0001
Variety^∗^isolate^∗^year	465.5933	15.5197	0.66	0.9088	9576.904	319.23	6.39	<.0001

Pr (>*F*): significant probability associated with the *F* statistic.

## Data Availability

Data are available on request.
